# Risky behaviors and road safety: An exploration of age and gender influences on road accident rates

**DOI:** 10.1371/journal.pone.0296663

**Published:** 2024-01-22

**Authors:** Dakota McCarty, Hyun Woo Kim

**Affiliations:** 1 Department of Urban Policy & Administration, Incheon National University, Incheon, South Korea; 2 Urban Science Institute, Incheon National University, Incheon, South Korea; Libyan Academy, LIBYA

## Abstract

Human behavior is a dominant factor in road accidents, contributing to more than 70% of such incidents. However, gathering detailed data on individual drivers’ behavior is a significant challenge in the field of road safety. As a result, researchers often narrow the scope of their studies thus limiting the generalizability of their findings. Our study aims to address this issue by identifying demographic-related variables and their indirect effects on road accident frequency. The theoretical basis is set through existing literature linking demographics to risky driving behavior and through the concept of “close to home” effect, finding that the upwards of 62% of accidents happen within 11km of a driver’s home. Using regression-based machine learning models, our study, looking at England, UK, explores the theoretical linkages between demographics of an area and road accident frequency, finding that census data is able to explain over 28% of the variance in road accident rates per capita. While not replacing more in-depth research on driver behavior, this research validates trends found in the literature through the use of widely available data with the use of novel methods. The results of this study support the use of demographic data from the national census that is obtainable at a large spatial and temporal scale to estimate road accident risks; additionally, it demonstrates a methodology to further explore potential indirect relationships and proxies between behaviors and road accident risk.

## 1. Introduction

Understanding and quantifying human behavior, especially in diverse and ever-changing populations, is a central challenge in behavioral sciences. This complexity arises from the myriad of external factors influencing individual actions across different timeframes [1]. A specific subset of this challenge exists in transportation, where human behavior directly correlates with road safety. Previous studies have underscored that risky behaviors are responsible for over 70% of road accidents; such behaviors include, but are not limited to, reckless driving, distracted driving, and driving under the influence or fatigue [2–5]. Given the significant influence these behaviors wield on road safety, enhancing our ability to estimate these behaviors could substantially improve road accident prediction models, shape more effective policies, and foster meaningful data generation.

However, the vast and diverse nature of any population presents methodological hurdles. Collecting data on every actor’s emotional state, intoxication level, and propensities to act in more risky manners is infeasible. In order to lessen these hurdles, researchers often narrow their scope to specific demographics, locations, or other controlled variables, facilitating data collection through surveys, in-car monitoring devices, or driving simulations [6]. While there have been impressive results, this narrowed scope invariably sacrifices the generalizability of the research to broader populations and areas which in itself can be considered a limitation.

To bridge this gap, our research aims to harness more widely available and generalizable variables to estimate these hard-to-observe behaviors on a larger scale. A promising avenue, as highlighted in previous literature, is the connection between demographic data and risky driving propensities. Additionally, another intriguing observation from the previous literature is the "close to home" effect associated with road accidents. With reports showing that 77% of accidents take place within 24 km of home and 33% happen within 1.6 km of home, showing additional statistics that roads within 11 km of home accounted for half of all travel and 62% of all accidents [7].

In this context, our study delves into the potential of leveraging census data to estimate road accident frequencies. Building on the established ties between demographic factors like age and gender and risky driving behaviors, with additional theoretical underpinnings from the close to home effect of accidents, this research hypothesizes that census data can be used to explain a notable amount of variance in road accident frequency. To further optimize our predictions and mitigate limitations frequent in statistical modeling of complex datasets, advanced regression-based machine learning techniques were employed in this study.

## 2. Literature review

### 2.1 Demographics and risky behaviors

The relationship between human behavior and road accidents has been robustly established in traffic safety research. Notably, over 70% of accidents are attributed to human behavior, underscoring its significance in this field [2, 3]. Such patterns of behavior provide not only an academic understanding but also avenues for potential enhancements in road safety.

Speeding, for instance, is a complex concern deeply interwoven with both individual and environmental variables. Factors such as age, sex, and the driver’s condition—alongside environmental conditions like road quality, traffic density, and weather—contribute to the framework of speed-related road safety [8, 9]. Precise modeling of these influences offers insights for potential interventions to address speed-related accidents.

Indeed, age and gender play pivotal roles in risky driving tendencies. Research consistently shows that younger male drivers often display more hazardous driving habits [10–12]. Men, in particular, tend to neglect traffic regulations, indulge in aggressive driving, and frequently overlook safety precautions, such as seatbelts [13, 14]. In contrast, while women generally exhibit more caution on the road, this is hypothesized to be associated with a quicker psychological maturity compared to men [15, 16]. Additionally, the inclination of males, especially those in their 30s, to breach road rules is concerning [17].

Young individuals, notably those aged 15–24, often manifest a combination of zeal and risk-taking, making them more inclined to dangerous driving behaviors [18]. Even though they are involved in fewer accidents—which could be due to the younger age bracket’s lower likelihood of possessing a driver’s license—the severity of their accidents is markedly higher. Conversely, older individuals, while typically more prudent drivers, are unfortunately more vulnerable to severe injuries, especially when pedestrians [19–21].

Further examination of risky behaviors sheds light on the concerning relationship between alcohol consumption and accident risks, as highlighted by Taylor et al. [22]. This connection has been reaffirmed in various global contexts, including Nepal and Nigeria [23, 24], and police records further emphasize alcohol’s significant role in both fatal and grievous injury incidents [25]. Alarmingly, the cognitive impairments caused by fatigue resemble those induced by alcohol, with particular demographics like younger drivers and shift workers being more susceptible [26]. For instance, the risk of a driver nodding off at 2:00 am is 50 times higher than at 10:00 am [27, 28].

In the contemporary context, distracted driving has evolved as a significant hazard. Numerous studies, utilizing tools like simulators and surveys, delve into the effects of various distractions, ranging from mobile phone usage to billboard distractions [29, 30]. Teenage drivers, with their higher tendency to use mobile phones and louder music, are particularly at risk, given their limited driving experience [31, 32]. Seasoned drivers, however, exhibit more restraint, suggesting that experience might reduce recklessness [33, 34]. Furthermore, emotional states and personality traits, such as risk propensity, can substantially alter driving behavior [35, 36], indicating a compelling confluence of psychology and road safety.

In summation, the literature paints a multi-faceted picture of risky driving behaviors, shaped by personal, environmental, and psychological dimensions. The nuanced interplay between demographics, specifically age and gender, with these risky behaviors is comprehensively summarized in Table 1.

**Table 1:** Summary of literature related to risky driving behavior and road accident risk.

Factor	Influence on Road Accident Risk	Studies
**Risky Behaviors**		
**Human Behavior**	Attributed to over 70% of road accidents; key factor in risk assessment	[2, 3]
**Vehicle Speed**	Influenced by human & environmental factors such as age, sex, vehicle capacity, road layout, weather	[8, 9]
**Alcohol Consumption**	Dose-response relationship; odds ratio for accidents rises with consumption.; decreased reaction speeds and attention; relationship with other behaviors	[22–25]
**Fatigue Driving**	Affects young age, shift workers, etc.; similar impact to drunk driving	[26–28]
**Distracted Driving**	Arises from cell phone use, music, etc.; higher risk for teenagers	[29–32, 37]
**Reckless Driving**	Includes high alcohol consumption; less likely in experienced drivers	[21, 22]
**Personality Traits**	Depression, anxiety linked to increased accidents; neuroticism elevates risk	[35, 36]
**Age and Gender**		
**Younger Drivers**	More prone to risky driving, fatal accidents; men more affected	[18–20, 38–40]
**Male Drivers**	More likely to ignore signs, speed, drive aggressively; more accidents due to driving style	[10–15, 17]
**Older Drivers**	Generally safer but more likely to be injured; pedestrians at higher risk	[41]

### 2.2 Close to home effect

The phenomenon of road accidents occurring closer to one’s residence has been previously discussed. One study delved into this aspect, revealing that accidents over-represented near homes predominantly occurred on low-speed (urban) roads and were more often attributed to lapses in attention than violations. Furthermore, such accidents were more frequent at minor intersections and mid-blocks than at major ones on these urban roads [41].

A study from the National Highway Traffic Safety Administration (NHTSA) reveal that a significant proportion of vehicular accidents occur in close proximity to one’s residence, with 52% of all accidents happening within an eight-kilometer radius and 69% within sixteen kilometers of home [42]. Complementing these findings, another research comprehensive data to validate the widely held belief about the prevalence of accidents near one’s home, finding that roads within an 11 km radius from home accounted for 62% of all accidents, with a strong association with alcohol and diverted attention. Together, these studies underscore the intricate interplay of familiarity, attention, and behavior in driving, suggesting that everyday routes might pose unexpected risks for drivers.

Based on this literature review, we can infer that younger, male drivers are most likely to exhibit more risky driving behaviors, thus increasing their odds allowing for further inference that they are most at risk of being in an accident. Additionally, these inferences paired with the statistical evidence that accidents are most likely to occur nearby one’s home, it is possible to further postulate that accident frequencies may be higher in areas which have higher densities of populations of whom are more likely to act more riskily, increasing their road accident risks. This suggest the potential theoretical linkages and backing of the viability of widely available and easily accessible census data to be used in road safety research.

## 3. Materials and methods

This study uses data from the UK Department of Transportation [43] for the year 2019. The choice of 2019 was based on it being the most recent year with a complete and normalized data set before the COVID-19 pandemic. Road accident deaths decreased significantly during the pandemic, with a 68% decrease in April 2020 compared to April 2019 [43]. Thus, to avoid data inconsistencies, 2019 was selected having a total of 106,981 accidents.

### 3.1 Study area and hexagonal gridding

The first step of the process was to generate an H3 hexagonal grid [44] with resolution level 8 (area = ~730m^2^) using the *h3pandas* Python package [45]. This resolution is comparable to the size of many of the United Kingdom’s, urban Lower Layer Super Output Areas (LSOAs), which typically comprise around 1,500 residents and 650 households [46], allowing for a more natural connection with the census data. This research has opted to use the hexagonal grid over other grid systems as they have a stronger ability to preserve spatial clustering patterns due to its equidistant nature [47].

After the hexagonal grid generation, census data was collected from the *Population estimates for the UK*, *England and Wales*, *Scotland and Northern Ireland*: *mid-2019* dataset [48] and then further interpolated according to Tobler’s pycnophylactic interpolation using Python and PySAL’s *tobler* package and the *area_interpolate* function within the *area_weighted* class [49]. Tobler’s pycnophylactic interpolation is a geographical data smoothing technique that preserves the total volume of data while estimating values between known data points. It is often used in fields like population density mapping, where it ensures that total population remains accurate, distributing densities across the landscape while creating a smooth spatial distribution [50].

The 106,981 accident data points were spatially aggregated into 34,554 hexagons using Python and *geopandas* and a basic count function to count the accidents falling within each of the respective hexagons [51]. To better target areas and minimize noise, a further sample of the data was taken only using with a minimum of 4 accidents in 2019, leaving a total of 7,537 hexagons making up the final sample size. Then, to calculate the accident rates (dependent variable), the accident counts for each hexagon were tallied and divided by the area’s total interpolated population.

When observing the demographic mosaic within this sample dataset, the male-to-female ratio, averages at 1.005 across the regions. Intriguingly, this ratio experiences notable fluctuations when dissected by age. The 10–19 years demographic witnesses a pronounced male predominance with a ratio peaking at 6.580, while the elderly segment, those above 65 years, leans towards a female majority with an average ratio of 0.850.

The age-wise population distribution further paints a comprehensive picture. The 20–34 years demographic emerges as the most populous, constituting roughly 22.85% of the inhabitants. Following closely are individuals aged between 35–49 years, making up 19.85% of the populace. The representation of both the nascent (under 10 years) and the senior (above 65 years) cohorts is commendable, with respective shares of 12.61% and 15.69%. This demographic equilibrium, illustrated vividly on the map, provides a foundational understanding of the study area’s populace, setting the stage for subsequent analyses. Table 2 highlights the variables used in the model along with their basic descriptive statistics.

**Table 2:** Variables for modeling of driver behavior and demographic features.

Feature	mean	std.	min.	max.
Accidents per capita	0.001	0.002	0.0001	0.056
Male to Female ratio	0.978	0.114	0.620	5.025
Male to Female ratio aged <10	1.068	0.178	0.461	2.802
% of population aged <10	0.110	0.029	0.005	0.408
Male to Female ratio aged 10–19	1.072	0.221	0.164	10.194
% of population aged 10–19	0.111	0.026	0.024	0.482
Male to Female ratio aged 20–34	1.064	0.456	0.339	20.894
% of population aged 20–34	0.160	0.068	0.041	0.772
Male to Female ratio aged 35–49	0.961	0.193	0.392	7.543
% of population aged 35–49	0.183	0.029	0.030	0.338
Male to Female ratio aged 50–64	0.975	0.115	0.621	4.041
% of population aged 50–64	0.214	0.040	0.018	0.364
Male to Female ratio aged > = 65	0.884	0.112	0.371	2.500
% of population aged > = 65	0.222	0.072	0.007	0.611

**Fig 1:**
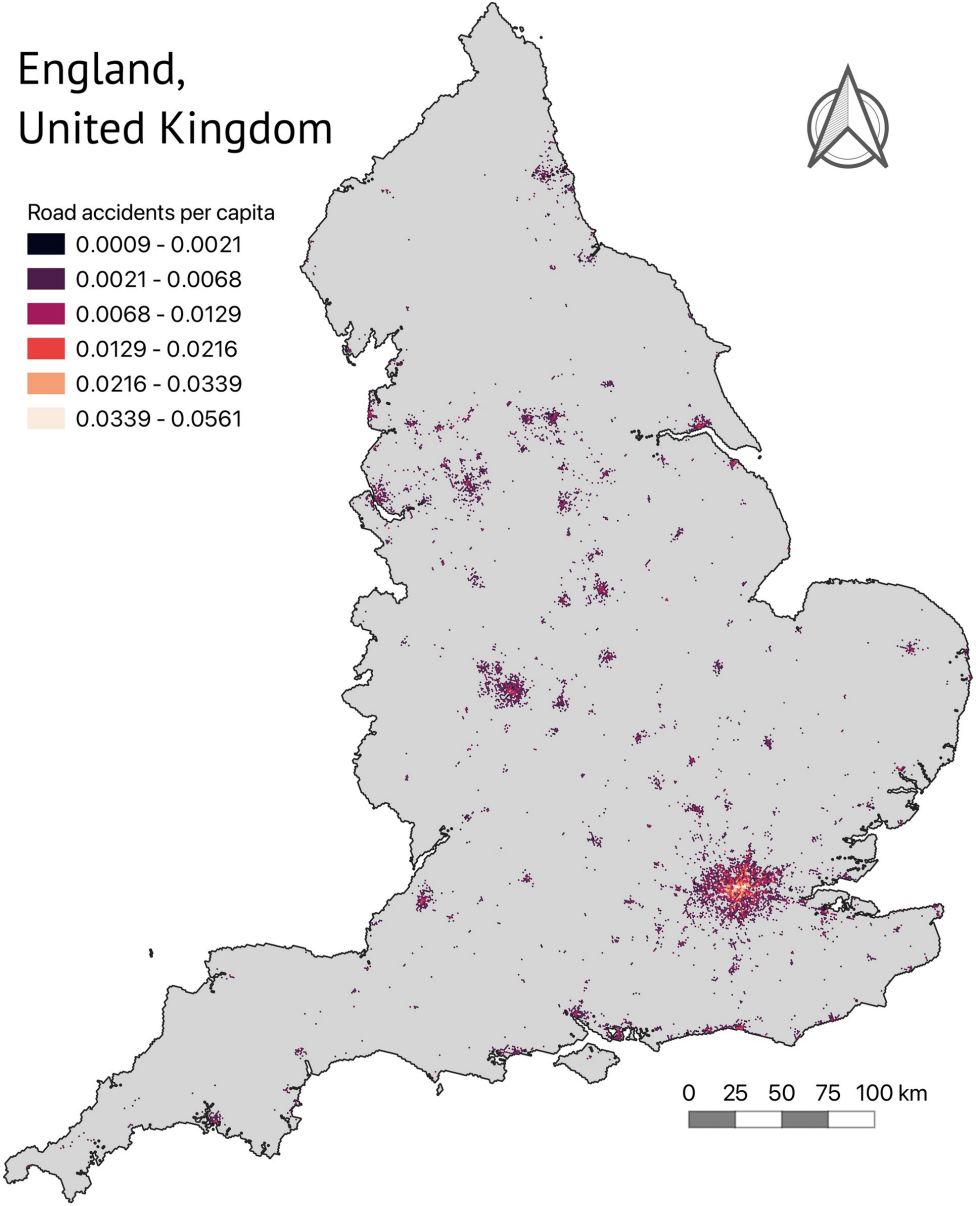
Accident rates per capita across England.

To better understand the data and spatial distributions of accident rates throughout England, Fig 1 illustrates the distribution of accidents in the study area.

The annual report underscores the predominance of behavioral causes, encompassing elements such as injudicious actions, driver errors, reactions, impairments, distractions, and inexperience. The multifaceted behavioral nature of road accidents in the study area is revealed through an analysis that identifies significant contributing factors (Table 3). Speeding emerges prominently, accounting for 32% of accidents, with an equal distribution of 7% each for exceeding speed limits and traveling too fast for conditions, and a concerning 18% due to driver carelessness or recklessness. This category is closely followed by driving under the influence or while fatigued, which includes 2% for fatigue, 6% for alcohol, and 2% for other drugs, summing up to 10% of the total accidents.

**Table 3:** Road accident behavioral causes by subcategory.

Category	Subcategory	Percentage (%)
**Speeding**	Exceeding Speed Limits	7
	Traveling Too Fast for Conditions	7
	Driver Carelessness or Recklessness	18
**Driving Under the Influence or Fatigued**	Fatigue Driving	2
	Alcohol Consumption	6
	Other Drugs	2
**Distracted Driving**	Using a Cell Phone	1
	Distractions within Vehicle	4
	Distractions Outside Vehicle	1
**Inexperience and Reckless Driving**	Inexperience	5
	Driving Too Slow for Conditions	1
	Other Subcategories (e.g., Following Too Close, Sudden Braking)	22
**Emotions**	Aggressive Driving	6
	Nervous, Uncertain, or Panic	1
**Total**		**83**

Distracted driving, while constituting a smaller percentage of 6%, is not to be overlooked, with specific contributions from cell phone usage, inside and outside distractions. Inexperience and reckless driving, on the other hand, play a more significant role, representing 28% of accidents. This includes 5% from inexperience, 1% from slow driving, and a substantial 22% from various subcategories like following too close and sudden braking. Emotional factors such as aggressive driving and feelings of nervousness add another 7%, making them an essential aspect to consider. Together, these categories provide a comprehensive view of up to 83% of recorded road accidents.

### 3.2 Modeling

To investigate the relationship between road accident rates and demographic characteristics, a variety of regression-based models were built using the variables from Table 2 as an input with the output being the expected accident rate per capita. In order to build the machine learning models, *PyCaret*, an open source AutoML tool, was used [52]. A major benefit to PyCaret is that it can produce a wide range of models in order to find what has the strongest performance.

Our PyCaret model was set up as follows:

setup(dataset, target=’Accidents per Capita’, normalize=True, normalize_method=’robust’, session_id=123)

In this setup, the dataset is passed (with our dependent and independent variables) with the target set to be “Accidents per Capita” and a *session_id* being passed as it acts as a seed allowing for easy replicability. Additionally, PyCaret was used to conduct normalization (by passing normalize=True as a parameter) on the dataset using the “robust” method (by passing the normalize_method=’robust’ parameter). Normalization is a technique often applied as part of data preparation for machine learning. The goal of normalization is to rescale the values of numeric columns in the dataset without distorting differences in the ranges of values or losing information. In the case of our set up, with the “robust” method, it scales and translates each feature according to the Interquartile range [53].

After running that code, the following PyCaret function was run to compare the performance of the produced models, setting the variable *best_model* to be the top performing model:

best_model = compare_models()

In total, this produced 18 separate models with their respective goodness-of-fit metrics. However, only the top six were further analyzed, with linear regression being used as a baseline (Table 4). In our case, the Extra Trees Regressor model outperformed the others and was selected as our *best_model*. It is important to note that while model hyperparameter tuning was attempted with PyCaret’s built in methods the model did not improve, leaving us to default to the best performing model.

**Table 4:** Machine learning model results.

Model	MAE	RMSE	R^2^	MAPE
Extra Trees Regressor	0.0024	0.0038	0.2872	0.4828
Light Gradient Boosting Machine	0.0023	0.0038	0.2670	0.4556
Random Forest Regressor	0.0024	0.0038	0.2584	0.4889
Gradient Boosting Regressor	0.0024	0.0039	0.2306	0.4714
K Neighbors Regressor	0.0025	0.0040	0.1810	0.4763
Bayesian Ridge	0.0026	0.0041	0.1582	0.5223
Linear Regression	0.0026	0.0041	0.1578	0.5237

To build our final model, the following code was utilized:

final_model = create_model(best_model)

This uses the PyCaret *create_model* function to further train the input model (our best_model, the Extra Trees Regressor) to obtain the final model. The results of this are shown below in Table 5 with the mean values highlighted. The following table shows that the model had a max R^2^ of 0.3587, a minimum of 0.2131, and a mean of 0.2872 after 10 folds.

**Table 5:** Results of Extra Trees Regressor model training.

Fold	MAE	MSE	RMSE	R^2^	MAPE
0	0.0025	0.0000	0.0042	0.3271	0.4669
1	0.0024	0.0000	0.0040	0.2907	0.4581
2	0.0021	0.0000	0.0030	0.3587	0.4507
3	0.0024	0.0000	0.0039	0.2131	0.5401
4	0.0022	0.0000	0.0032	0.2760	0.4842
5	0.0024	0.0000	0.0036	0.2479	0.4903
6	0.0024	0.0000	0.0037	0.2971	0.4798
7	0.0027	0.0000	0.0050	0.3072	0.4880
8	0.0022	0.0000	0.0031	0.3364	0.4867
9	0.0024	0.0000	0.0037	0.2177	0.4829
Mean	0.0024	0.0000	0.0038	0.2872	0.4828

The Extremely Randomized Trees (Extra Trees) algorithm offers an improvement on the ensemble-based approach of decision trees. Its uniqueness lies in the method it employs to determine split points. The conventional decision tree’s splitting criterion for regression tasks, is as follows. Given a dataset, the objective is to find a feature and a threshold that minimizes the variance of the target variable *y* within the resulting partitions.

The variance of *y* for a given set *S* is:

$$Var\left(S\right)=\frac{1}{\left|S\right|}\sum_{i\epsilon S}{\left(y_{i}-{\bar{y}}_{S}\right)}^{2}$$ (1)

Where:

• |*S*| is the number of samples in set *S*.

• ${\bar{y}}_{S}$ is the mean value of the target variable in set *S*.

For a feature *f* and a threshold *t*, the dataset is split into two subsets *S*_*left*_ and *S*_*right*_. The variance reduction sought is:

$$VarianceReduction\left(f,t\right)=Var\left(S\right)-\left(\frac{\left|S_{left}\right|}{\left|S\right|}Var\left(S_{left}\right)+\frac{\left|S_{right}\right|}{\left|S\right|}Var\left(S_{right}\right)\right)$$ (2)

In this equation, the goal is to find the feature and threshold that maximizes this variance reduction.

The key departure of Extra Trees from traditional decision trees is in the selection of the threshold *t*. Instead of considering every possible split and choosing the best one, Extra Trees picks a random threshold for each feature under consideration. The best among these randomly selected splits (in terms of variance reduction) is then chosen to split the node.

Formally, given a set of features *F* for each feature *f* ϵ *F*, a random threshold t_f_ is selected from its range. The variance reduction is computed for each such (*f*, *t*_*f*_) pair, and the pair that offers the most variance reduction is chosen for the split.

Like Random Forest, Extra Trees builds an ensemble of such trees. The final prediction for a given input is the average of the predictions from all the trees in the ensemble:

$$\hat{y}=\frac{1}{T}\sum_{i=1}^{T}y_{i}$$ (3)

Where:

• *T* is the number of trees in the ensemble.

• *y*_*i*_ is the prediction of the *i*^*th*^ tree.

This contributes to a few notable advantages over other models—By eschewing the exhaustive search for the best split, Extra Trees often achieves faster training times; The model often exhibits higher bias due to the randomness but compensates with a reduction in variance, making it more robust to overfitting; And the randomness leads to more diverse trees, which aids in the ensemble’s overall performance, especially when dealing with intricate datasets.

The results of the Extra Trees Regressor will be further explored in the coming sections to explain the results more deeply and how they relate to the previous literature as well as the aims and scope of this research.

## 4. Results and discussion

The Extra Trees Regressor model showed a Mean Absolute Error (MAE) of 0.0024, Root Mean Square Error (RMSE) of 0.0038, R-squared (R^2^) value of 0.2872, and Mean Absolute Percentage Error (MAPE) of 0.4828. The R^2^ may seem low, but it is important to consider the complexity of the problem being addressed. In this case, the model only considers demographic features, such as age and gender percentages and ratios, to predict a complex phenomenon as accident rates. With that consideration, the R^2^ score is reasonable given the limited input being able to explain nearly 29% of the variance of a complex problem.

Additionally, it is worth noting that the model is not intended to be used as a standalone predictive tool, but rather as a tool to establish a link between road accident rates and demographic characteristics, built through previously discussed theoretical linkages. Furthermore, the MAE and RMSE values are relatively small and within a comparable range, indicating that the model is accurate in its predictions. The acceptable MAPE score is also a good indicator of model performance. In summary, while the value of R^2^ may seem low, it is reasonable given the scope of the model and the results of other evaluation metrics suggest that the Extra Trees Regressor is an effective with establishing a link between demographic characteristics and road accident rates.

**Fig 2:**
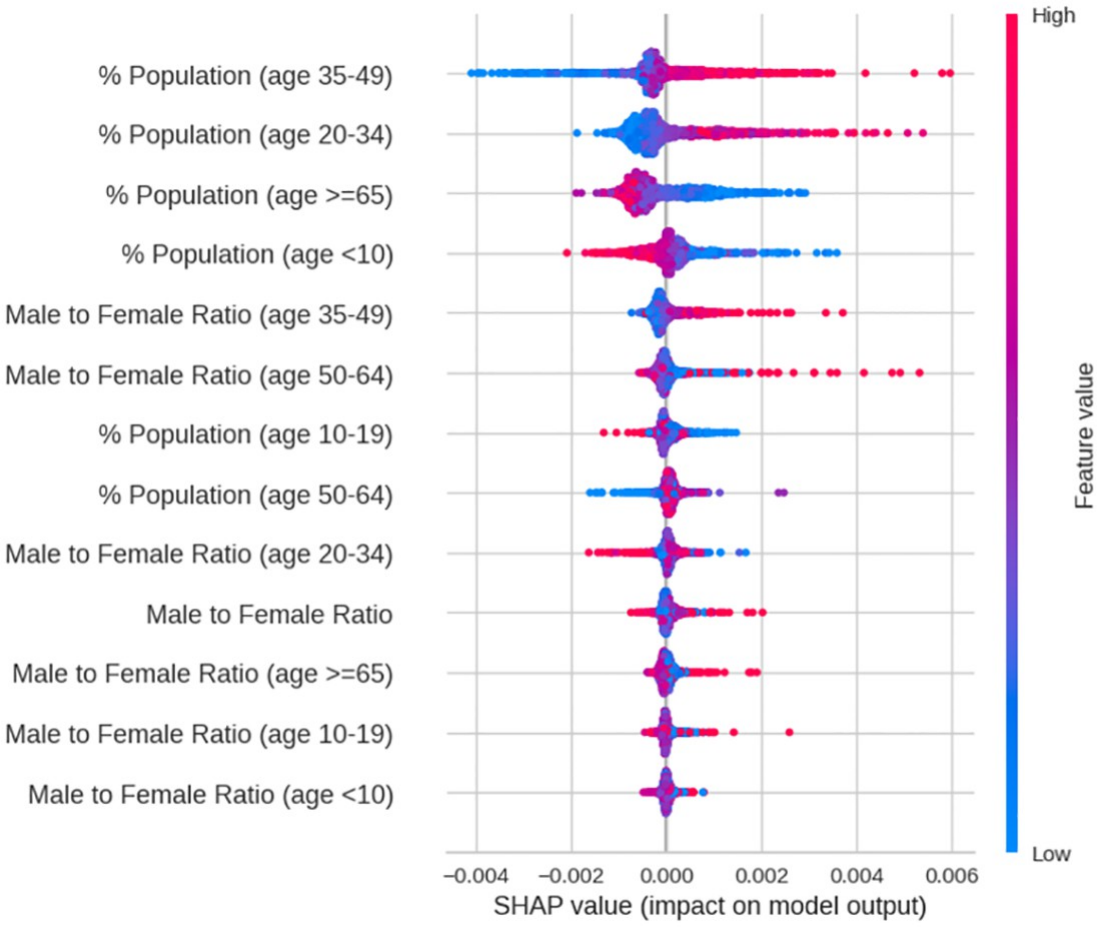
SHAP distribution plot for demographics.

The SHAP (SHapley Additive exPlanation) values have been employed in Fig 2 to provide a more precise interpretation of the results. SHAP is a mathematical technique used to explain the predictions of machine learning models based on concepts of game theory. Each feature can be calculated to help to understand the predictions of the model [54].

When interpreting a SHAP chart, the features are arranged in descending order of importance, each point in the plot represents a sample point of the data, and the colors (a gradient from red to blue) represent the value of the sample point. For instance, in the case of "% of Population (age 35–49)", the red-er the point indicates a higher percentage of the population aged 35–49, while the bluer the point indicate a lower percentage. Furthermore, the points are distributed along the plot with 0.0 on the x-axis indicating no major relationship between the dependent variable and the features; points to the left of the 0 have a negative effect on the prediction, while points to the right have a positive impact.

The SHAP bee swarm plot (Fig 2) generated by the Extra Trees Regressor model reinforces the trends found during the literature analysis. Specifically, the SHAP plot indicates that road accident rates increase when there is a greater proportion of people aged 35–49 than those aged 20–34 and decrease as the elderly population increases. The gender of the population is also a factor, and road accidents are more common in areas where there are more men between 35 and 49 years of age. Although the trends for the other features are less clear, they do learn towards aligning with previous research.

**Fig 3:**
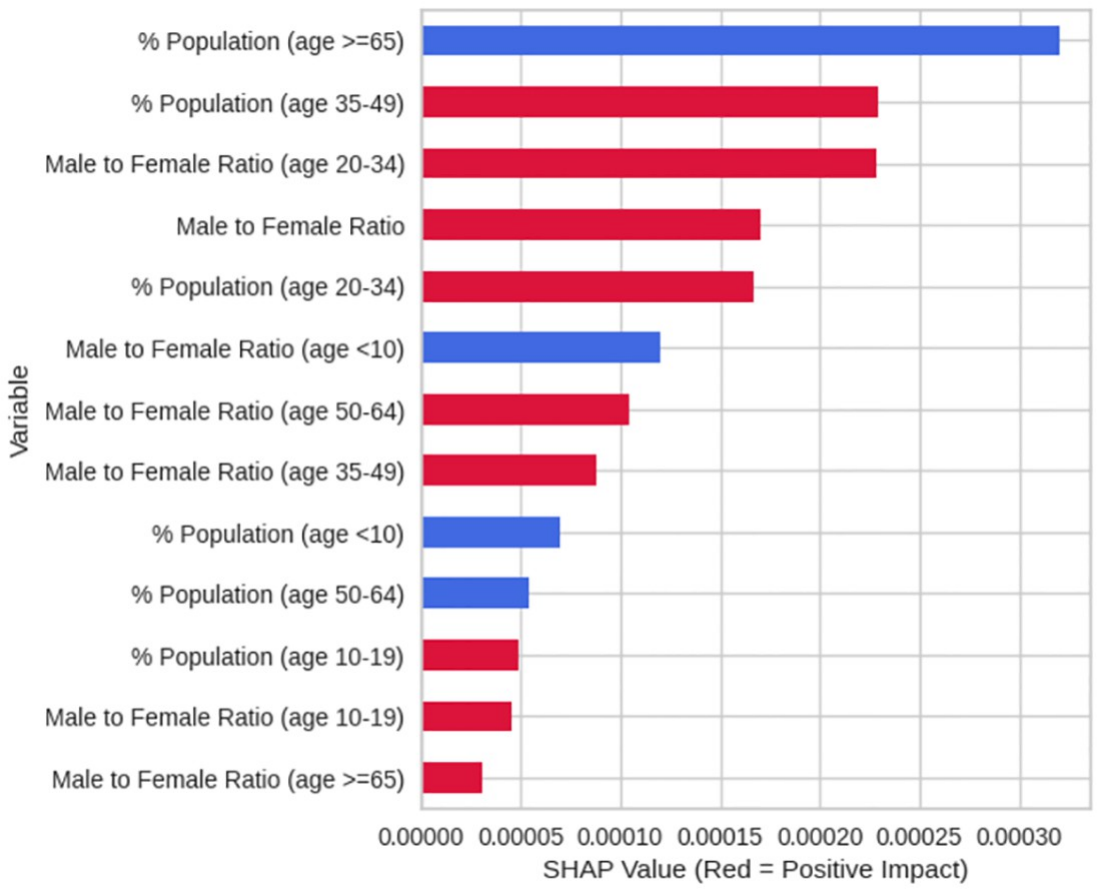
Mean Absolute SHAP values and their impact.

Another SHAP plot was produced to better understand the relationship that each feature had. Below, Fig 3, shows the mean absolute SHAP value for each feature, in order of effect, as well as the average direction that the feature pushed the direction with red indicating an increase in road accident rates and blue indicating a decrease. This plot is useful as it gives a clear representation of the data.

Fig 3 shows that there is a higher percentage of the population being over the age of 65 decreasing road accidents, and a near equal importance for percentage of the population being aged 35 to 49 and the ratio of males to females aged 20 to 34 with both increasing road accident rates. Overall, this plot shows that in areas with more males there is a higher rate of road accidents, and in areas with adolescents and the elderly having lower rates of road accidents.

Overall, the top three findings from analyzing the results of the model are that as the percentage of the population aged 65 or older increases, the average predicted ’Accidents per Capita’ decreases. This suggests that areas with a larger proportion of older residents tend to have fewer accidents per capita, according to the model. Additionally, that the average predicted ’Accidents per Capita’ increases slightly as the percentage of the population aged 35–49 increases, but then it decreases after the percentage of that population gets to a certain point. This suggests that there might be an optimal range of the percentage of the population aged 35–49 where the number of accidents per capita is highest. Outside this range, the number of accidents per capita decreases, according to the model. Then, as the average predicted ’Accidents per Capita’ increases as the male-to-female ratio in the 20–34 age group increases. This suggests that areas with a higher male-to-female ratio in this age group tend to have more accidents per capita, according to the model aligning well with previous literature.

It is important to note that the presence of these trends does not necessarily mean that there is a direct correlation between the demographics of an area and road accident rates; nor are the results surprising or novel in their own rights as research has proven across a range of fields that there are connections between demographics and risky behaviors as well as risky behaviors and road accident risks. However, the methodology and data used to further quantify these linkages is novel demonstrating that the linkages found in previous, in-depth, case studies on driver’s behavior and demographics holds true even with more generalized data (i.e., census data). These results can inform a range of areas from more efficient planning and resource allocations of EMS and other accident-related services to insurance premiums, to more informed accident prediction models.

## 5. Implications

The implications of this research extend to a broad spectrum of stakeholders, from urban planners and policymakers to researchers in the field of road safety. Our study underscores the pivotal role of demographic data, especially from the national census, in illuminating patterns and predictors of road accident frequencies. The findings further validate previous research finding that human behavior, deeply influenced by demographic variables, plays a significant role in road accidents, accounting for a substantial proportion of such incidents. However, our findings bring additional novelty through the use of more general and available data, whereas previous research utilized surveys, in-car sensors, and other methods that are unfeasible at a large scale as they involve identifying the demographics of every individual car.

With the rapid proliferation of data and modeling techniques, the importance of establishing robust theoretical links between datasets and the variables they predict cannot be overstated. Machine learning models, while powerful, lack innate abilities to decipher the intricacies of data. The responsibility of this deciphering then falls on the researchers. Our work seeks to fulfill this by drawing upon abundant existing literature, notability in behavioral and psychological sciences as well as the "close to home" effect, which reveals that a staggering 62% of accidents occur within 11km of a driver’s residence and over 70% of accidents are caused by behavioral factors.

By utilizing regression-based machine learning models, we’ve successfully elucidated the connection between an area’s demographic profile and road accident frequency in England, UK. The findings suggest that census data can explain more than a quarter of the variance in per capita road accident rates. This not only reaffirms the utility of widely accessible data in echoing intricate patterns observed in more granular studies but also introduces innovative methodologies to analyze potential indirect relationships.

For urban planners, our research paves the way for more precise road safety models by integrating census data. This integration could amplify their model’s accuracy and facilitate well-informed decisions. Policymakers, equipped with insights from our study, can develop targeted risk maps. These maps can spotlight regions with higher propensities for accidents, paving the way for proactive interventions such as public awareness campaigns or heightened law enforcement presence.

Furthermore, the ability to identify areas with a higher concentration of "risky" populations can help optimize emergency response strategies. By strategically deploying Emergency Medical Services (EMS) in these zones, not only can accidents be preempted, but the aftermath of incidents can be more effectively managed.

## 6. Conclusion

This study is anchored in the belief that census data can elucidate variations in accident rates, building upon established relationships between risky driving, demographics, and road accidents with the "close to home" phenomenon, which highlights the propensity for accidents to occur near one’s residence, providing a theoretical supports the utilization of census data for this analysis.

While behavioral sciences have made considerable advances, challenges persist in acquiring granular and expansive driver data. By intertwining census data with the “close to home” principle, our research surmounts some of these challenges. The resulting model, though open to further enhancement, explains 28.7% variance in road accidents despite only having limited features. Reinforcing insights from prior studies, our results suggest the theoretical influence of driver behavior and demographics on accident rates is possible. Future research could enrich this model by integrating overlooked factors like infrastructure, weather conditions, and others that have been found to influence road accident rates.
